# Synthesis of Single-Crystal Graphene on Copper Foils Using a Low-Nucleation-Density Region in a Quartz Boat

**DOI:** 10.3390/mi12101236

**Published:** 2021-10-12

**Authors:** Kaiqiang Yang, Jianlong Liu, Ruirui Jiang, Yubin Gong, Baoqing Zeng, Zichuan Yi, Qingguo Gao, Jianjun Yang, Feng Chi, Liming Liu

**Affiliations:** 1 School of Electronic Science and Engineering, University of Electronic Science and Technology of China, Chengdu 610054, China; 201811022515@std.uestc.edu.cn (K.Y.); liujianlong@uestc.edu.cn (J.L.); 201711040120@std.uestc.edu.cn (R.J.); ybgong@uestc.edu.cn (Y.G.); 2 Zhongshan Branch of State Key Laboratory of Electronic Thin Films and Integrated Devices, Zhongshan Institute, University of Electronic Science and Technology of China, Zhongshan 528402, China; yizichuan@zsc.edu.cn (Z.Y.); gqg@hust.edu.cn (Q.G.); sdyman@uestc.edu.cn (J.Y.); chifeng@semi.ac.cn (F.C.)

**Keywords:** single-crystal graphene, nucleation density, quartz boat, copper foils, copper pockets

## Abstract

The nucleation of graphene at different locations in the quartz boat was studied, and the lowest nucleation density of graphene in the quartz boat was found. The nucleation density of graphene is the lowest at the bottom of the quartz boat near the gas inlet side. Based on the above results, a simple and reproducible way is proposed to significantly suppress the nucleation density of graphene on the copper foil during the chemical vapor deposition process. Placing the copper foil with an area of 1.3 cm × 1 cm in the middle of the bottom of the quartz boat or further back, and placing two copper pockets in front of the copper foil, an ultra-low nucleation density of ~42 nucleus/cm^2^ was achieved on the back of the copper foil. Single-crystal monolayer graphene with a lateral size of 800 μm can be grown on the back of copper foils after 60 min of growth. Raman spectroscopy revealed the single-crystal graphene to be in uniform monolayers with a low D-band intensity.

## 1. Introduction

Graphene is composed of two-dimensional carbon atoms assembled in a hexagonal structure, akin to a honeycomb, and has exceptional chemical stability, superior mechanical stability, and very high thermal conductivities and electronic conductivities [1,2,3]. Because of the above excellent properties, graphene has potential applications in ultra-high-speed electronics [4], flexible transparent conductive films [5,6], solar cells [6], separation membranes [7], and transmission electron microscopy (TEM) imaging [8]. Graphene was first isolated by mechanical exfoliation from Highly Oriented Pyrolytic Graphite (HOPG) [1]. Although various methods have been used to grow graphene on a solid surface such as HOPG, electrochemical exfoliation, epitaxy growth, and chemical reduction of graphene oxide [9], chemical vapor deposition (CVD) on metal foils is believed to be a promising method to produce large-area mono- or few-layer graphene [10]. However, a polycrystalline structure and a high density of domain/grain boundaries in typical graphene grown by CVD on metal foils cause variable electronic properties in different areas and hinder the applications of graphene in devices [11,12]. Graphene grain boundaries (GGBs), which form in regions where graphene domains with different orientations merge, are observed as line defects in graphene film and result in the degradation of the thermal, electrical, and chemical properties of graphene [13,14,15,16,17,18]. Accordingly, it is necessary to study the growth and preparation of single-crystal graphene. For example, the electrical performance of graphene is degraded by the additional scattering centers induced by GGBs, causing a reduction in the carrier mobility and conductivity of graphene [19].

It is still a challenge to grow large-size single-crystal graphene. There are two ideas to improve the size of single-crystal graphene, which are the single-seed method and the multi-seed method [20]. The key of the single-seed method is to control the nucleation density of graphene. Maintaining lower surface roughness of the substrate and reducing the supply of carbon are two main methods to reduce the nucleation density of graphene. One classic method is the copper pocket method [21], which is used to explore the distribution of methane partial pressure in a quartz boat in our work. The multi-seed method requires the preparation of special substrates. The orientation dependence of graphene on the substrate surfaces, which is vital to the aligned nucleation of graphene islands, is mainly determined by the interaction and the lattice-matching degree between graphene and the underlying substrates. To date, the epitaxial growth of graphene has been reported on the surfaces of some special substrates such as Cu(111), Ni(111) [22], Au(111) [23], Pt(111) [24], and CuNi alloy [25,26]. Among these, Cu(111) plane is highly advantageous for the aligned nucleation and growth of graphene domains, as its hexagonal lattice symmetry matches the honeycomb lattice of graphene well (lattice mismatch ≈4%), thereby enabling the epitaxial growth of graphene, upon which the graphene islands can demonstrate simultaneous growth and seamless merging [27,28,29].

In this paper, the nucleation of graphene at different locations in the quartz boat isexplored through three groups of comparative experiments. Based on the results of these experiments, we report a simple and reproducible way to significantly suppress the nucleation density by placing the copper foil with an area of 1.3 cm × 1.0 cm in the middle of the bottom of the quartz boat, or further back, and placing two copper pockets in front of the copper foil. Nucleation density of graphene on the back of copper foils can be significantly reduced and an ultra-low nucleation density of ~42 nucleus/cm^2^ was achieved. After increasing the growth time to 60 min, single-crystal monolayer graphene with a lateral size of 800 μm can be grown on the back of copper foils under the optimized growth condition. In addition, the Raman study indicates that the single-crystal graphene synthetized by our method has a very low D-band intensity on the silicon dioxide grown on Si wafers, which is of high quality.

## 2. Materials and Methods

### 2.1. Synthesis of Graphene

**Figure 1:**
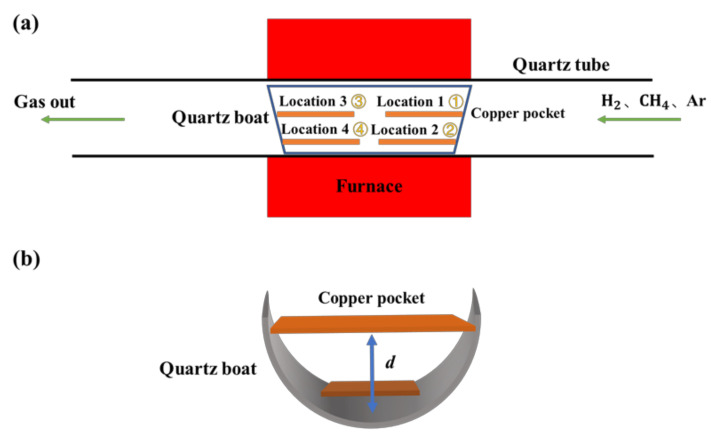
(**a**) Schematic illustrations of the distribution of four regions in the quartz boat in the transverse direction and the CVD system. (**b**) Schematic illustrations of the copper pockets placed in the quartz boat.

Graphene was synthesized by the low-pressure CVD in a tube furnace. The copper foils were folded into copper pockets, and a Cu pocket was made by first bending a copper foil (99.8%, Alfa Aesar, Shanghai, China, annealed, uncoated, #46365) and then crimping or pressing the three open edges carefully by using metal tweezers [30]. The copper pockets were placed flat in the quartz boat, which was located in the center of the quartz tube of a CVD system. The whole interior of the quartz boat is divided into four regions. These four regions are referred to as location 1 to location 4, respectively. Horizontally, the locations of the copper pockets in the quartz boat are shown in Figure 1a. Vertically, the locations of the copper pockets in the quartz boat are shown in Figure 1b,d is the distance from the copper pocket to the bottom of the quartz boat. Three groups of experiments were completed for exploring the nucleation of graphene in different regions of the quartz boat. Two copper pockets placed in different locations in the quartz boat were added to the CVD system in each experiment. In the first group of experiments, two copper pockets were located in location 2 and location 3 of the quartz boat, respectively. In the second group of experiments, two copper pockets were located in location 1 and location 4 of the quartz boat, respectively. In the third group of experiments, two copper pockets were located in location 2 and location 4 of the quartz boat, respectively. The same parameters were used in the three experiments: furnace temperature 1030 °C Ar:H_2_:CH_4_ = 50:50:0.5 (in the unit of sccm), and growth time of 45 min. After studying the nucleation of graphene at different locations in the quartz boat, a new growth mode was used. A piece of copper foil with an area of 1.3 cm × 1.0 cm was prepared and then placed at the middle position of the bottom of the quartz boat. The distance between the copper foil and the bottom of the quartz boat was 0.5 cm, and the center of the copper foil was about 4 cm away from the tail of quartz boat (the quartz boat was 10 cm long, 4 cm wide, and 2 cm high). At the same time, two copper pockets were placed in front of the copper foil in order to further reduce the methane flow at the bottom of the quartz boat, as shown in Figure 6a. Finally, the quartz boat with copper foils was placed into the quartz tube and the growth test was carried out in the center of the heating furnace. The growth parameters were as follows: furnace temperature 1030 °C, Ar:H_2_:CH_4_ = 50:50:0.5 (in the unit of sccm), and growth time of 60 min. Each group of experiments was repeated multiple times, until the results were stable.

### 2.2. Transfer of Graphene

A thin film of polymethyl methacrylate (PMMA) (HF-kejing, Hefei, China, 6 wt.% in anisole) was spin-coated onto the graphene/Cu (600 r/min for 6 s then 3000 r/min for 40 s). The etching of Cu usually takes 3 h. Then, the PMMA/graphene/Cu thin film was placed into 30 mg/mL ammonium persulfate ((NH_4_)_2_S_2_O_8_) aqueous solution (Aladdin, Shanghai, China, purity of 98%, AR). After etching, the PMMA/graphene was rinsed in DI water several times. The PMMA/graphene was dried in a desiccator and then placed on the SiO_2_/Si substrate. The PMMA layer was dissolved using hot acetone (Guangzhou, China, purity of 99.5%, AR).

### 2.3. Characterization

After finishing the growth of graphene, the copper pockets and copper foils were opened to copper foils, then the copper foils were placed on the hot table at 200 °C for 5 min. The oxidized surfaces of copper foils were observed under the optical microscope. Due to the effect of the oxidation resistance of graphene [31], the areas covered by graphene were not oxidized, and the areas not covered with graphene were oxidized to copper oxide. The areas covered by graphene were brighter than other areas. Therefore, we could directly observe the shape and size of single-crystal graphene on copper foils by optical microscope. The diameter and nucleation density of single-crystal graphene under different growth conditions are the average values of repeated experiments. Scanning electron microscopy (TESCAN, Brno, Czech Republic) was also used to characterize the morphology of graphene. Raman spectroscopy (FuXiang, Shanghai, China) was used to characterize the number and quality of graphene layers.

## 3. Results and Discussion

**Figure 2:**
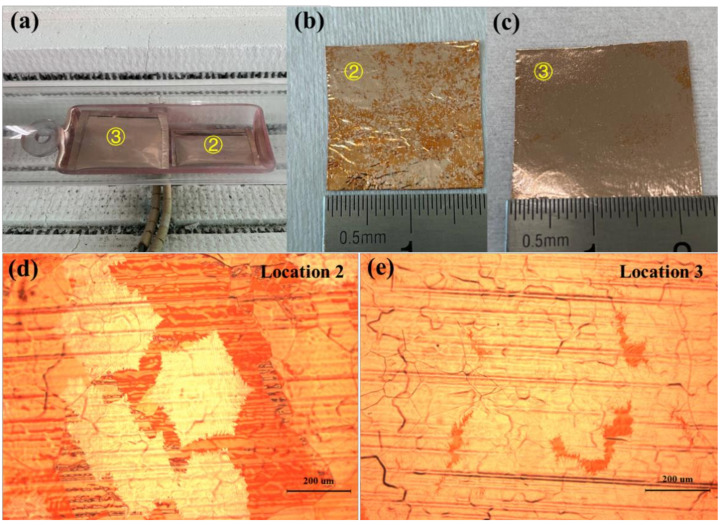
(**a**) Optical picture of the two copper pockets placed in the quartz boat in the first experiment. (**b**,**c**) Optical picture of the inner surfaces of the copper pockets at location 2 and location 3 after oxidation treatment. (**d**,**e**) Optical micrograph of the inner surfaces of the copper pockets at location 2 and location 3 under 100-times magnification.

In the first group of experiments, the copper pockets were placed as shown in Figure 2a. Location 2 and location 3 are described as the bottom of the quartz boat near the gas inlet side and the top of the quartz boat near the gas outlet side in the following, respectively. As shown in Figure 2b,c, the nucleation density of graphene on the inner surface of the copper pockets at location 2 was lower than that on the inner surface of the copper pockets at location 3 after growing for 45 min. The inner surface of the copper pockets at location 2 was not completely covered by graphene, the distribution of nucleation points of graphene was scattered, and there were many places without graphene. Figure 2d is the optical micrograph of the inner surface of copper pockets at location 2 under 100-times magnification. Single-crystal graphene grains could be observed under the microscope. The shape of single-crystal graphene was approximately hexagonal, and the average diameter of the hexagonal grains was 335 μm. Figure 2e is the optical micrograph of the inner surface of copper pockets at location 3 under 100-times magnification. Single-crystal graphene was not observed, and the graphene regions were combined with each other; only a few areas were covered with copper oxide (dark red area). Here, we use the reported graphene visualization methods [31], where the graphene/copper sample is oxidized in air and results in a reflective contrast between the oxidized copper in the exposed regions and the unexposed copper covered by graphene. Through the above observation, we found that the nucleation density of graphene on the bottom of the quartz boat near the gas inlet side was lower than that in the top of the quartz boat near the gas outlet side with the copper pockets method.

**Figure 3:**
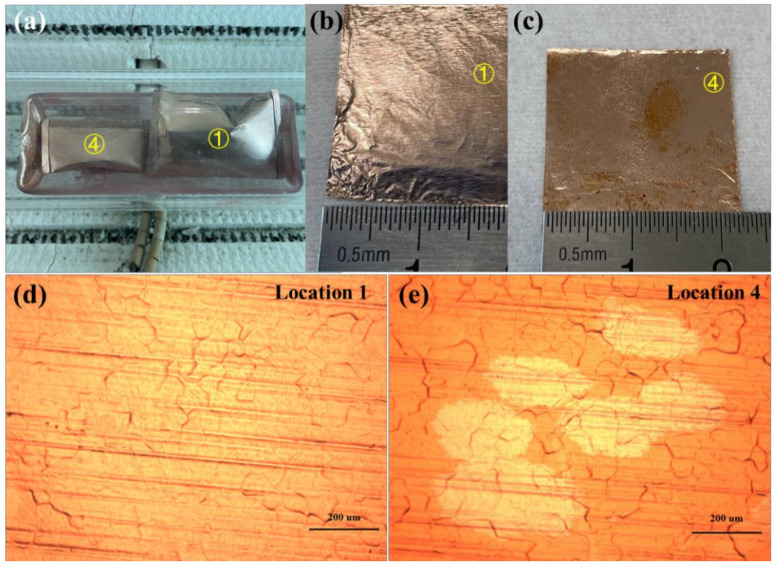
(**a**) Optical picture of the two copper pockets placed in the quartz boat in the second group of experiments. (**b**,**c**) Optical picture of the inner surfaces of the copper pockets at location 1 and location 4 after oxidation treatment. (**d**,**e**) Optical micrograph of the inner surfaces of the copper pockets at location 1 and location 4 under 100-times magnification.

In the second group of experiments, the copper pockets were placed as shown in Figure 3a. Location 4 and location 1 are described as the bottom of the quartz boat near the gas outlet side and the top of the quartz boat near the gas inlet side in the following, respectively. As shown in Figure 3b,c, the inner surface of the copper pockets at location 1 was completely covered by graphene, but there were still some areas on the inner surface of the copper pocket at location 4 which were not covered by graphene. Figure 3d is the optical micrograph of the inner surface of the copper pockets at location 1 under 100-times magnification. Single-crystal graphene was not observed, and the graphene regions were combined with each other; the substrate was completely covered with graphene. Figure 3e is the optical micrograph of the inner surface of the copper pockets at location 4 under 100-times magnification. Single-crystal graphene grains could be clearly observed under the microscope. The regions of single-crystal graphene were independent, with an average size of 215 μm. We found that the nucleation density of graphene in the bottom of the quartz boat near the gas outlet side was lower than that in the top of the quartz boat near the gas inlet side from the second group of experiments.

**Figure 4:**
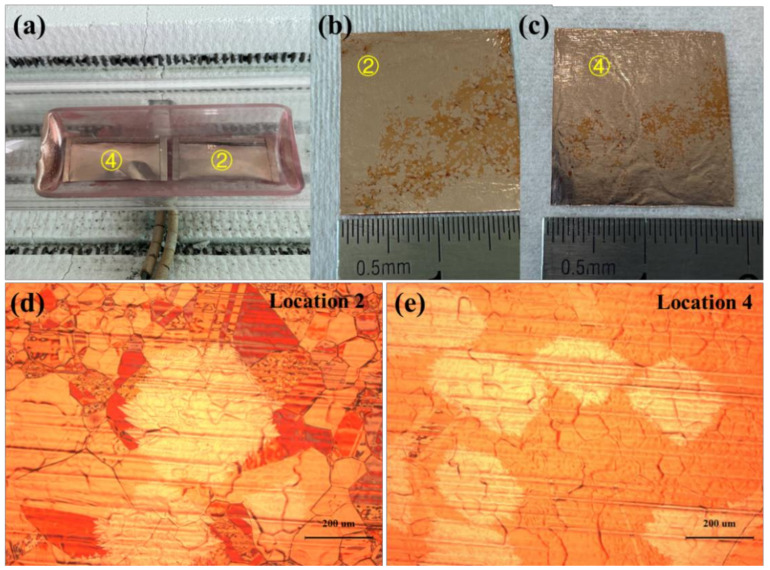
(**a**) Optical picture of the two copper pockets placed in the quartz boat in the third group of experiments. (**b**,**c**) Optical picture of the inner surfaces of the copper pockets at location 2 and location 4 after oxidation treatment. (**d**,**e**) Optical micrograph of the inner surfaces of the copper pockets at location 2 and location 4 under 100-times magnification.

In the third group of experiments, two copper pockets were placed as shown in Figure 4a. As shown in Figure 4b,c, the inner surfaces of the copper pockets at location 2 and location 4 were not completely covered by graphene, and the distribution of the graphene regions was scattered. Comparing Figure 4b,c, we also find that the area not covered by graphene on the inner surface of the copper pockets at location 2 was larger than that at location 4, and the nucleation density of graphene on the inner surface of the copper pockets at location 2 was lower than that at location 4. Figure 4d is the optical micrograph of the inner surface of the copper pockets at location 2 under 100-times magnification. Single-crystal graphene grains could be observed under the microscope. The shape of single-crystal graphene was approximately hexagonal, and the average diameter of the hexagonal grains was 467 μm. Figure 4e is the optical micrograph of the inner surface of the copper pockets at location 4 under 100-times magnification. Single-crystal graphene grains could be observed under the microscope. The average size of single-crystal graphene was 221 μm. Summing up the third group of experiments, we found that the nucleation density of graphene in the bottom of the quartz boat near the gas inlet side was lower than that in the bottom of the quartz boat near the gas outlet side.

**Figure 5:**
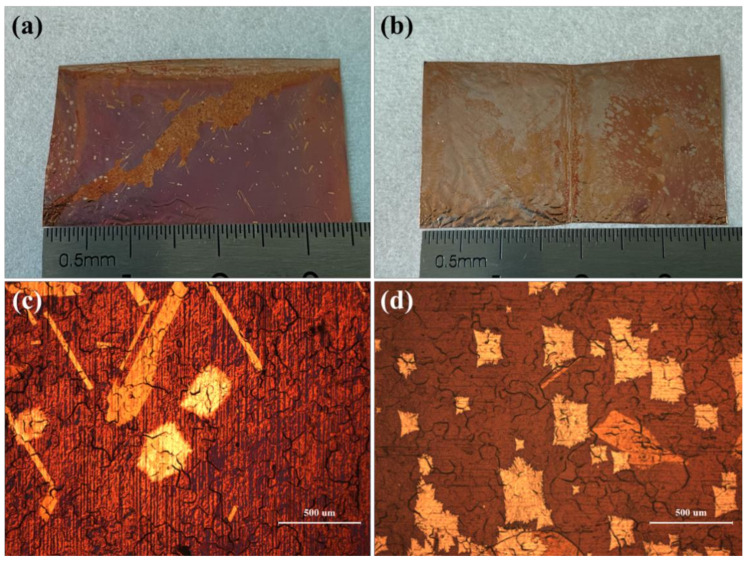
(**a**,**b**) Optical picture of the back of the copper pockets (outer surfaces) at location 2 and location 4 after oxidation treatment. (**c**,**d**) Optical micrograph of the back of the copper pockets (outer surfaces) at location 2 and location 4 under 50-times magnification.

In our experiments, we also observed that the single-crystal graphene can grow on the back of the outer surfaces of the copper pockets (the side near the bottom of the quartz boat) and the nucleation density of graphene was very low. There were only about 102 nucleation sites of graphene in the area of 3.5 cm × 2 cm, and the nucleation density of graphene is 27 nucleus/cm^2^. Figure 5a is an optical picture of the back of the outer surface of the copper pockets at location 2. The nucleation points of graphene were sparsely distributed on the copper foil. Figure 5c is the optical micrograph of the back of the outer surface of the copper pockets at location 2 under 50-times magnification. Single-crystal graphene grains could be observed under the microscope. The shape of single-crystal graphene was approximately hexagonal, and the average diameter of the hexagonal grains was 398 μm. Figure 5b is an optical picture of the back of the outer surface of the copper pockets at location 4. The lighter areas are graphene grains (the color contrast between graphene and oxidized substrate is not obvious). The area covered by graphene was larger than that at location 2. The nucleation density of graphene was also higher than that at location 2. Figure 5d is the optical micrograph of the back of the outer surface of the copper pockets at location 2 under 50-times magnification. Single-crystal graphene grains could also be observed under the microscope. The diameter of single-crystal graphene was in the range of 50–360 μm. In this group of experiments, the nucleation density of graphene in the bottom of the quartz boat near the gas inlet side was lower than that in the bottom of the quartz boat near the gas outlet side, which also supports the conclusion of the third group of experiments.

The above four groups of experiments were carried out multiple times in each group. The distribution of the nucleation density of graphene at four locations in the quartz boat was obtained after stable results appeared. We found that the nucleation density of graphene at the bottom of the quartz boat (location 2 or location 4) was lower than that at the top (location 1 or location 3), and the closer to the gas inlet side (location 1 or location 2), the lower the nucleation density of the graphene in the copper pockets method. In the copper pockets method, the growth of single-crystal graphene depends on the smooth inner surface of the copper pockets during the growth process. The smoothness of the inner surface is created by the dynamic balance of the evaporation and deposition of copper inside copper pockets [32]. In addition, the closed structure of copper pockets also makes the partial pressure of methane in the copper pockets very low. Low methane partial pressure can effectively reduce the nucleation density of graphene, which makes it possible to prepare large-size single-crystal graphene [21]. Therefore, the different nucleation density of graphene in the quartz boat should be related to the partial pressure of methane in the quartz boat. In the growth stage, the partial pressure of methane at the bottom was lower than that at the top, and that at the location near the gas inlet side was lower than that near the gas outlet in the quartz boat. 

**Figure 6:**
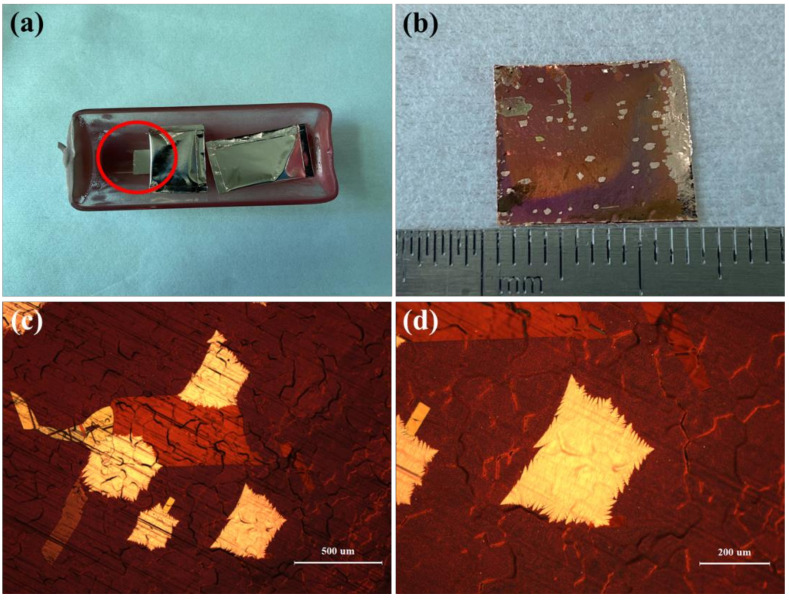
(**a**) Optical picture of the copper foil placed in the quartz boat in the experiment. (**b**) Optical picture of the back of the copper foil with single-crystal graphene. (**c**) Optical micrograph of single-crystal graphene on the back of the copper foil under 50-times magnification. (**d**) Optical micrograph of single-crystal graphene on the back of the copper foil under 100-times magnification.

Based on the above results, we proposed a simple and reproducible way to synthesize single-crystal graphene. A copper foil with an area of 1.3 cm × 1.0 cm was placed on the middle of the bottom of the quartz boat, or further back, and two copper pockets were placed in front of the copper foil, as shown in Figure 6a. The copper foils being placed in the middle of the bottom of the quartz boat, or further back, was based on our previous experiments, where the partial pressure of methane was low and the nucleation density of graphene was low. Two copper pockets were placed in front of the copper foils to further reduce the methane rate at the bottom of the quartz boat. The copper pockets played a role in shielding, so as to reduce the partial pressure of methane at the bottom of the quartz boat, and the nucleation density of graphene on the copper foils was also lower. In many experiments, we found that as long as the copper foils that were cut to about 1 cm^2^ (at this time, the back of copper foils were about 0.5 cm away from the bottom of the quartz boat) were placed at the bottom of quartz boat, and two copper pockets were in front of this, single-crystal graphene could be stably obtained on the surfaces of copper foils. Figure 6b is an optical picture of the back of the copper foil with single-crystal graphene. After 45 min of growth, there were 58 nucleation points of graphene on the back of the copper foil (the side facing the bottom of the quartz boat). The nucleation density of graphene was about 42 nucleus/cm^2^ under this condition. Figure 6c is the optical micrograph of the back of the copper foil under 50-times magnification. Figure 6d is the optical micrograph of the back of the copper foil under 100-times magnification. Single-crystal graphene grains could be observed under the microscope. The average diameter of single-crystal graphene is 429 μm.

**Figure 7:**
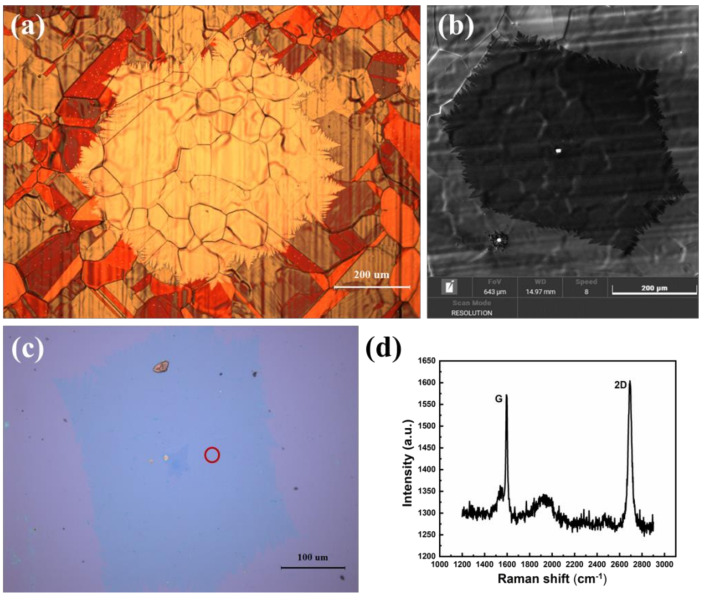
(**a**) Optical micrograph of single-crystal graphene on the back of the copper foil under 100-times magnification. (**b**) SEM image of single-crystal graphene domains on the copper foil. (**c**) Optical micrograph of single-crystal graphene transferred onto silicon dioxide grown on Si wafers under 100-times magnification. (**d**) The Raman spectra of the single-crystal graphene transferred onto the silicon dioxide grown on Si wafers.

By placing copper foils on the bottom of the quartz boat (two copper pockets were placed in front of the copper foils, as shown in Figure 6a), a low nucleation density of graphene was obtained at the back of the copper foil. When growth time was increased to 60 min, single-crystal graphene of a diameter about 800 μm was obtained, under other unchanged growth conditions. Figure 7a is the optical micrograph of single-crystal graphene on the back of the copper foil under 100-times magnification. The average diameter of single-crystal graphene was about 800 μm. Figure 7b is the SEM image of graphene domains on the copper foil, and the edge of single-crystal graphene presents a fractal structure resembling a snowflake. Single-crystal graphene domains on the copper foils were transferred onto dielectric substrates such as silicon dioxide, using a poly(methyl methacrylate) (PMMA)-assisted method for Raman characterizations. Figure 7c is the optical micrograph of single-crystal graphene on silicon dioxide grown on Si wafers (the thickness of silicon dioxide is 300 nm) under 100-times magnification. Figure 7d shows the Raman spectra of the single-crystal graphene transferred onto the silicon dioxide grown on Si wafers. The I_2D_/I_G_ intensity ratio was ~1.2, and there was no obvious D peak. High-quality graphene was obtained by our growth and transfer method.

## 4. Conclusions

In conclusion, the nucleation of graphene at different locations in the quartz boat was explored by setting up several comparative experiments. We found that the nucleation density of graphene at the bottom is lower than that at the top, and that at the location near the gas inlet side is lower than that near the gas outlet in the quartz boat. We also found that the single-crystal graphene can grow on the back of outer surfaces of copper pockets (the side facing the bottom of the quartz boat). The single-crystal graphene with a diameter of 800 μm can be obtained directly on copper foils by our method after growing for 60 min. Raman spectroscopy revealed the single-crystal graphene to be in uniform monolayers with a low D-band intensity. The single-crystal graphene prepared in our work can be used in many kinds of 2D electronics, such as flexible electrodes and flexible field effect transistors, because of its ultra-high carrier mobility and excellent flexibility.
